# Electrical brain networks before and after transcranial pulsed shockwave stimulation in Alzheimer’s patients

**DOI:** 10.1007/s11357-024-01305-x

**Published:** 2024-08-27

**Authors:** Lars Wojtecki, Celine Cont, Natalie Stute, Anastasia Galli, Christina Schulte, Carlos Trenado

**Affiliations:** 1 Departmemt of Neurology and Neurorehabilitation, Hospital Zum Heiligen Geist, Academic Teaching Hospital of the Heinrich-Heine-University Duesseldorf, Von-Broichhausen-Allee 1, 47906 Kempen, Germany; 2 Institute of Clinical Neuroscience and Medical Psychology, Medical Faculty, Heinrich Heine University, Duesseldorf, Germany; 3 Max Planck Institute for Empirical Aesthetics, Frankfurt Am Main, Germany

**Keywords:** Alzheimer’s disease, Coherence, Tsallis entropy, Power, Cross-frequency-coupling, Electroencephalogram, ultrasound, shock waves, Non-pharmacological, intervention

## Abstract

Alzheimer’s disease (AD) is a neurodegenerative disorder that dramatically affects cognitive abilities and represents the most common cause of dementia. Currently, pharmacological interventions represent the main treatment to deal with the symptoms of AD; however, alternative approaches are readily sought. Transcranial pulse stimulation (TPS) is an emerging non-invasive neuromodulation technique that uses short, repetitive shockwaves with the potential to provide a wide range of vascular, metabolic, and neurotrophic changes and that has recently been shown to improve cognitive abilities in AD. This exploratory study aims to gain insight into the neurophysiological effect of one session of TPS in AD as reflected in electroencephalographic measures, e.g., spectral power, coherence, Tsallis entropy (TE), and cross-frequency coupling (cfc). We document changes in power (frontal and occipital), coherence (frontal, occipital and temporal), and TE (temporal and frontal) as well as changes in cfc (parietal-frontal, parietal-temporal, frontal–temporal). Our results emphasize the role of electroencephalographic measures as prospective markers for the neurophysiological effect of TPS.

**Supplementary information:**

The online version contains supplementary material available at 10.1007/s11357-024-01305-x.

## Introduction

Alzheimer’s disease (AD) is a progressive neurodegenerative disorder characterized by the presence of amyloid plaques and neurofibrillary tangles [1], which lead to decline in cognitive functions such as memory, speech, visuospatial processing, and executive functions [2], which critically influences the life’s quality of AD patients.

Focusing on treatments for AD, pharmacological interventions include cholinesterase inhibitors and antagonists to N-methyl-d-aspartate [3] as well as lecanemab and aducanumab, which target the protein beta-amyloid to help reduce amyloid plaques [4, 5]. Nevertheless, the spectrum of possible mechanisms giving place to AD and the fact that some medical options are not indicated in certain patients or patients who suffer from progression despite optimal treatment makes it necessary to consider alternative treatments. In this respect, brain stimulation techniques, such as transcranial magnetic stimulation (TMS), deep brain stimulation (DBS), and transcranial electrical stimulation (tES), stand out. Whereas the latter might influence the clearance of proteins when applied alternating current stimulation (tACS) with Gamma-frequencies [6], the first two might be more focal and target specific [7]. For the usefulness of TMS, there is already sham-controlled evidence even in combination with electrophysiological or imaging biomarkers [8]. For tES in the form of transcranial direct current stimulation (tDCS), there is further evidence for cognitive-behavioral improvement and functional connectivity in combination with cognitive training [9].

Sonification/sound-based therapies are non-invasive stimulation techniques that are currently clinically investigated with preliminary—partly sham-controlled evidence—in a variety of neuropsychiatric conditions, such as pain, dementia, movement disorders, psychiatric conditions, epilepsy, disorders of consciousness, and developmental disorders [10].

Low-intensity focused ultrasound (FUS) is an emerging non-invasive therapeutic technology that relies on the use of sound waves to target brain regions with high specificity and without the need for incision or radiation [11]. With regard to AD, previous studies addressing the effect of FUS in mice models of dementia reported improvement of cognitive abilities [12], while studies targeting the hippocampus or substantia nigra in AD patients reported an improvement of cognitive and motor scores with good safety data [13]. Transcranial pulsed stimulation (TPS) is another sound-based technique different from ultrasound that provides short, repetitive shockwaves through a neuro-navigated device. Recent AD studies utilizing TPS reported scarce side effects and improvement in the Alzheimer’s disease Assessment Scale (ADAS) and ADAS cognitive scores [14], and improvement of depression scores as reflected in the Becks Depression Inventory (BDI-II) accompanied by effects in functional connectivity (FC) after one session of TPS [15]. TPS induced neuroplasticity changes up to 1 week after the last stimulation within a 3-week experimental longitudinal protocol [16]. Moreover, a recent study reported the effectiveness of TPS in reducing depressive symptoms in adults with major depressive disorder [17]. This highlights the expanding interest and clinical use of TPS worldwide. Among positive TPS features that have been emphasized are good spatial precision in stimulating brain regions with the possibility to reach deeper brain structures in comparison to other non-invasive brain stimulation techniques (TMS and tDCS) and adaptability of stimulation parameters, which leads to individual and targeted patient treatments. Challenges include lack of proper stimulation protocols and deeper understanding on the neurophysiological effect of stimulation as well as the interaction between sound waves generated by TPS and tissue.

In the present exploratory study, our aim was to gain insight into brain network changes after the first session of TPS as reflected in electroencephalographic measures such as spectral power, cross-frequency coupling (cfc), coherence, and Tsallis entropy (TE). Based on previous reports indicating significantly lower FC between the left frontal orbital cortex and the right anterior insula after TPS [15], we expected changes particularly in brain cognitive networks involving frontal cortical areas. Nevertheless, strictly speaking, we had a priori no definite hypothesis on the neurophysiological effect of TPS due to the novelty of the technique. Consequently, our study particularly targets the formulation of independent data-inspired hypotheses that could guide the experimental design of future TPS studies addressing specific AD patient cohorts.

## Methods

### Patients

Ten Alzheimer and TPS treatment–naïve patients (two female and eight male; age, 69.2 ± 7.1 years) were recruited for EEG analysis from the Department of Neurology and Rehabilitation at Hospital zum Heiligen Geist Kempen. Patients varied in the severity of cognitive symptoms: three patients with mild, five with moderate, and two with severe impairment.

Inclusion criteria for clinical indicated TPS treatment were at least Alzheimer’s clinical syndrome, defined by gradual progressive change in memory function (using the Mini-Mental Status Examination (MMSE) as screening tool for severity score) and impairment of activity of daily living for more than 6 months. In vivo evidence from cerebrospinal fluid analysis (CSF) and/or MRI scans were used for the National Institute on Aging and Alzheimer’s Association (NIA-AA) criteria, which categorizes the underlying pathological processes using biomarkers [1]. These biomarkers are grouped into ß amyloid deposition, pathological tau, and neurodegeneration (AT(N)), which can be detected in imaging and biofluids (see Table 1 for patient characteristics). Eight patients were defined as Alzheimer’s continuum, seven of them with Alzheimer’s disease (AD) and one with Alzheimer’s disease and concomitant suspected non-Alzheimer’s pathological change. Two patients were simply defined as having Alzheimer’s clinical syndrome due to a lack of biomarker and one as having Alzheimer’s clinical syndrome with non-Alzheimer’s pathological change. Exclusion criteria were relevant intracerebral pathologies (including vascular lesions Fazekas > 2) unrelated to Alzheimer’s disease, non-compliance with the protocol, blood clotting disorders, oral anticoagulation, corticosteroid treatment in the last 6 weeks, pregnancy, breastfeeding, or epilepsy.

**Table 1:** Baseline demographic, clinical, neuropsychological, and stimulation characteristics of patients considered in this study (*n* = 10). *M* male, *F* female, cognitive impairment according to MMSE: mild, 26–20; moderate, 19–10; severe: < 10, *AD* Alzheimer’s disease

ID	Age (year)	Sex	Cognitive impairment	Language spoken/handedness	Biomarker category/diagnosis	Number of TPS Impulses / Area stimulated
1	74	M	Severe	German/left	A + T + (N) + /AD	1004 precuneus, temporal
2	77	M	Moderate	German/right	AD syndrome without biomarkers tested	2004 precuneus, temporal
3	59	M	Moderate	German/right	A + T-(N) + ^a^	2001 precuneus, temporal
4	60	M	Moderate	German/right	A + T + (N) + /AD	6003 precuneus, parietal, frontal
5	65	M	Moderate	German/right	A + T + (N) + /AD	3000 precuneus, temporal, parietal, frontal
6	61	F	Mild	German/right	A + T + (N) + /AD	3001 frontal
7	74	M	Severe	German/right	AD syndrome without biomarkers tested	3000 precuneus, parietal, frontal
8	74	F	Moderate	German/right	A + T + (N) + /AD	6002 precuneus, parietal, frontal, temporal
9	67	M	Mild	German/right	A + T-(N) + ^b^	6000 precuneus, parietal, frontal, temporal
10	72	M	Mild	German/right	A + T + (N) + /AD	6000 precuneus, parietal, frontal, temporal

^a^Alzheimer’s and concomitant suspect non-Alzheimer’s pathological change

^b^Alzheimer’s clinical syndrome with non-Alzheimer’s pathological change

This EEG study was conducted in accordance with the Ethics Committee of the regional Medical Chamber (Ärztekammer Nordrhein, Nr. 2021137). Patients signed a written consent for participation.

### Study protocol

**Fig. 1:**
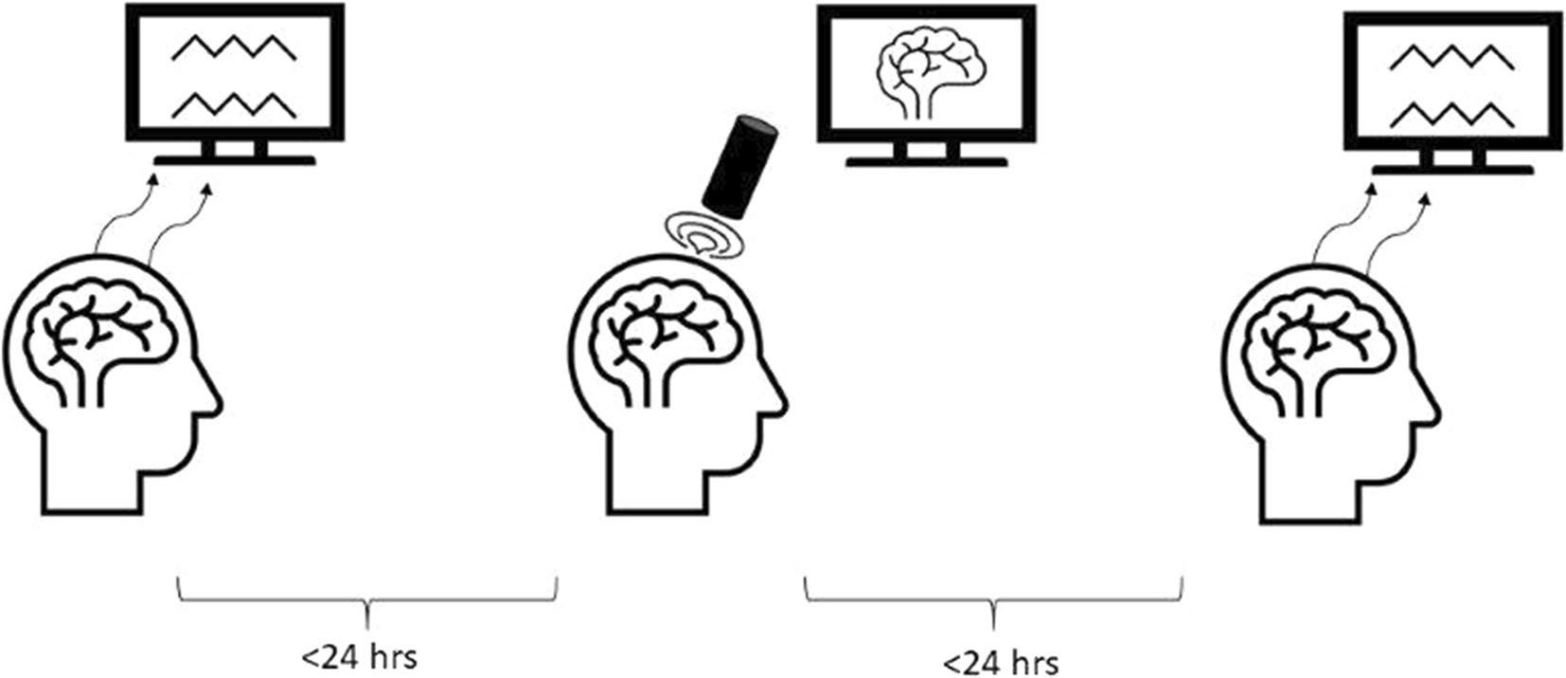
Protocol. EEG from 21 channels during resting state with predominantly eyes closed (at least 10 min) was performed directly or within 24 h before and after the first TPS session for each patient. TPS was applied with MR-neuronavigation and took around 45 min with navigation calibration

EEG from 21 channels during resting state with predominantly eyes closed (at least 10 min) was recorded directly or within 24 h before and after the first TPS session for each patient. TPS was applied with MR-neuronavigation and took around 45 min with navigation calibration (Fig. 1).

### TPS

We made use of the Neurolith© TPS device from Storz Medical, which allows neuronavigation using individual 3D T1 isometric voxel MRI scans. The treatment protocol was 4 Hz, 0.20 mJ/mm^2^ by default. The stimulation protocol was similar to Beisteiner et al. [18], including the bilateral frontal cortex, bilateral lateral parietal cortex, and extended precuneus cortex. In difference to Beisteiner et al. [18], also the bilateral temporal cortex was included in our protocol. To gain experience concerning tolerability and safety, the number of impulses and the areas was stepwise increased after the first session in the pilot patients.

See Table 1 for a list of number of stimulation pulses and areas stimulated in the first session for each patient. Each session took around 45 min with navigation calibration.

### EEG recording

EEG (Nihon-Koden 1100/1200) was recorded from 21 channels (10/20 system) during resting state with predominantly eyes closed (at least 10 min) directly or within 24 h, before and after the first TPS session for each patient. The recording sampling rate was 200 Hz and channel impedances were kept in the range 0–20KΩ. Ear clips (A1/A2) served as the reference. A filter between 0.3 Hz and 80 Hz was applied.

### EEG analysis

### Preprocessing

The following preprocessing steps were applied in the specified order: (1) EEG data was read, screened for quality check, and exported to EEGLAB format by using Brainstorm. (2) Artifacts corresponding to eye blinks and facial muscle activity were visually screened and manually rejected based on established criteria, via EEGLAB, by experienced EEG specialists among us. (3) EEG was referenced to the average reference (MATLAB). (4) Data was high-pass filtered at 0.1 Hz and notch filtered at 50 Hz to remove line disturbances (MATLAB). Only artifact-free segments were considered for further analysis. The length of such segments was in average 12.62 ± 4.99 min. Preprocessing steps were performed by using the open-source software Brainstorm and EEGLAB as well as customized scripts in MATLAB Version 9.12.0 (R2022a), Natick, Massachusetts: The MathWorks Inc., 2022. The MATLAB functions used for steps 3 and 4 are provided in the supplementary materials.

### Regions of interest

We considered the following cortical areas of interest: occipital (O1, O2), frontal-complete (Fp1, Fp2, F4, F3, F8, F7, and Fz), fronto-polar (Fp1, Fp2), temporal (T3, T4), and parietal (P3, P4, Pz). We focused on the frontal region (frontal-complete) concerning the effects of AD on cognitive abilities. Note that we considered a subset of frontal electrodes (Fp1 and Fp2) (fronto-polar) as it has been reported in relation to cognitive abilities for instance in studies demonstrating prefrontal EEG slowing, synchronization, and ERP latency in relation to pre-dementia stages in AD [19].

### Spectral analysis

Power spectrum was calculated by using fast Fourier transform with 0.39-Hz resolution and Hanning window (size of 512). We extracted spectral power from scalp regions of interest by averaging the power of channels corresponding to a specific region at a specified frequency band. We considered the following frequency bands: delta (1–4 Hz), theta (4–8 Hz), alpha (8–14 Hz), beta (14–31 Hz), gamma (31–80 Hz), theta-alpha (4–14 Hz), alpha–beta-gamma (8–80 Hz), and beta-gamma (14–80 Hz).

### Coherence

We estimated coherence between regions of interest as follows:

$$Co{h}_{X,Y}(f)=\frac{{\left|{\text{S}}_{\text{X},\text{Y}}\left(f\right)\right|}^{2}}{|S{P}_{X}\left(f\right)||S{P}_{Y}\left(f\right)|}$$

Here, ${S}_{X,Y}(f)$ denotes the cross-spectrum for the EEG average signal of regions $X$ and $Y$ at a given frequency $f$ and $S{P}_{X}$ and $S{P}_{Y}$ denote the respective spectral power $SP$ [20].

### Tsallis entropy

We estimated the entropy of EEG data by using a well-known measure, e.g., TE, under the assumption that the variance of an EEG event is directly proportional to its probability of occurrence and that EEG events can be captured by bins of information. For the calculation of TE, we considered the method proposed in [21]. Specifically, TE is given by

$$\widehat{H}\left(Y\right)=1-\frac{{\sum }_{i,j}^{M}{\widehat{{S}_{ij}}}^{2}}{{S}^{2}}$$

where *M* is the total number of data bins, ${\widehat{{S}_{ij}}}^{2}$ is the variance of each interval, and $\frac{{S}^{2}}{M}$ is an estimator of the time series variance ${\sigma }^{2}$. In particular, we employed bins with 100 equally spaced lengths between 1 and 500 points for the calculation. The average entropy value across bins was used as the estimated TE for each channel. We reported grand average TE across regions for each patient.

### Cross-frequency coupling

Cfc represents a measure of interaction between oscillations at different frequency bands, which may reflect synchronization of neural assemblies. In particular, we estimated cfc by using Canolty’s modulation index (MI), which has been shown to be less affected by noisy data compared to other techniques. Specifically, MI is given by

$$MI=\left|\frac{1}{N}\sum_{n=1}^{N}w\left[n\right]\right|$$

Here $w\left[n\right]={A}_{{f}_{1}}\left[n\right]{e}^{i*{\varnothing }_{{f}_{2}}\left[n\right]}$, where ${A}_{{f}_{1}}$ denotes the amplitude of the analytic signal representation of a time series $X$ at the modulated frequency range ${f}_{1}$ and ${\varnothing }_{{f}_{2}}$ denotes the phase of the analytic signal representation of $X$ at the modulating frequency range ${f}_{2}$. We adopted as modulating frequency range [1, 10 Hz] and modulated frequency range [20, 85 Hz] as implemented in [22]. The code used for the calculation of cfc is available in the following repository (https://github.com/AJQuinn/cfc).

### Statistical analysis

As the assumption of normality was violated (Kolmogorov–Smirnov), non-parametric tests were utilized. We made use of the Wilcoxon signed-rank test (paired samples) with a significant level of 5% to compare parameters between conditions pre and post TPS stimulation. The effect size was estimated by using $r=\frac{Z}{\sqrt{n}}$, where $Z$ is the approximate test statistic and $n$ is the number of pairs in the sample [23]. Statistical analysis was performed by using the software IBM SPSS Statistics (Version 25, IBM Software, Business and analytics, Armonk, NY, USA), Statistics Toolbox MATLAB, and the calculator for effect sizes [24].

## Results

**Fig. 2:**
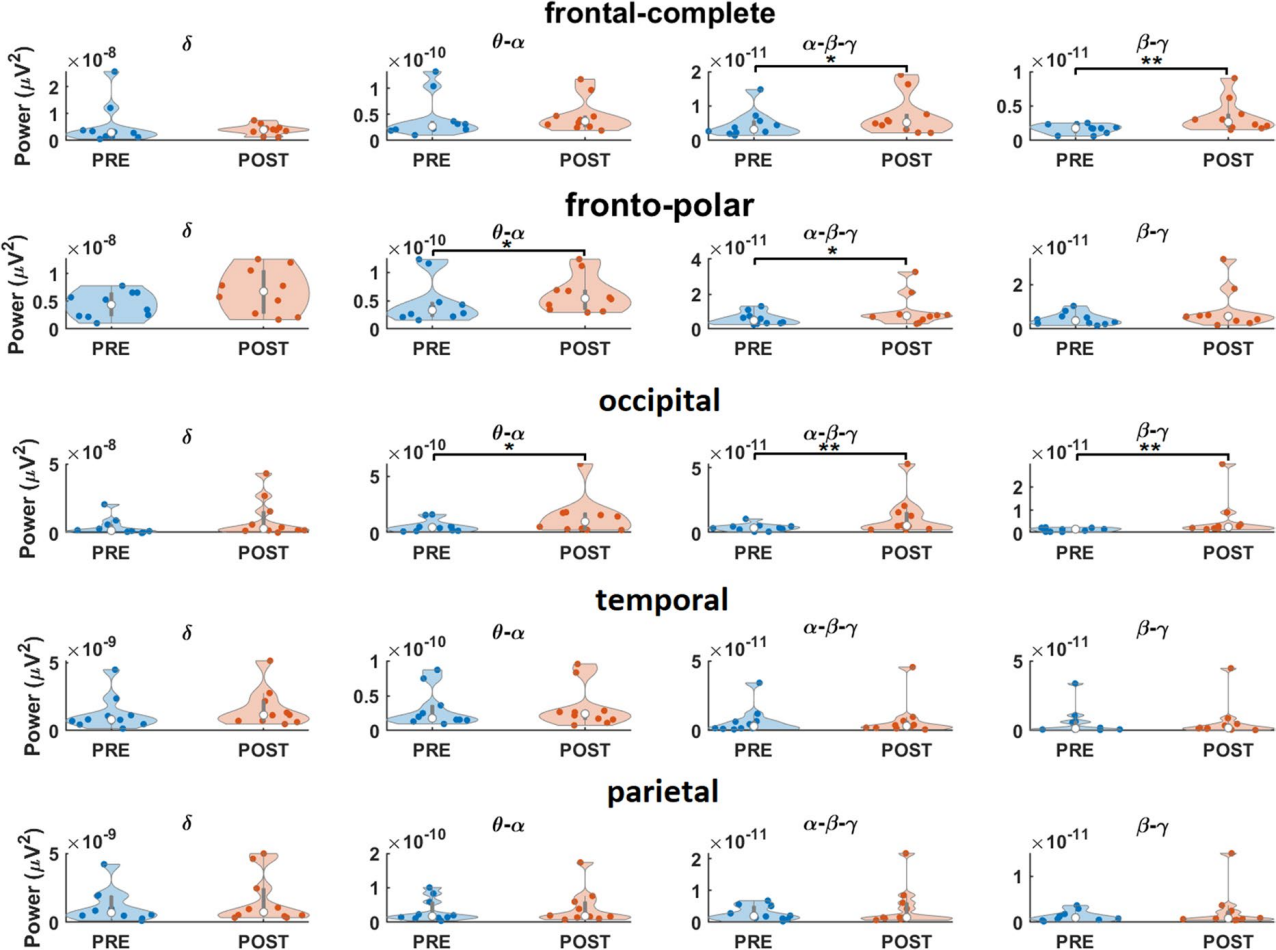
Effect of one TPS session on EEG power. Individual effects (across channels) at frontal-complete, fronto-polar, occipital, temporal, and parietal regions are marked as significant differences (**) and statistical trends (*)

With regard to power spectrum, Wilcoxon signed-rank test revealed a significant difference between condition pre- and post-TPS at region occipital in beta-gamma (*Z* =  − 2.4973, *p* = 0.013, η^2^ = 11.895, *r* = 0.7897), frontal-complete in beta-gamma (*Z* =  − 2.4973, *p* = 0.013, η^2^ = 11.895, *r* = 0.7897), and occipital in alpha–beta-gamma (*Z* =  − 2.5992, *p* = 0.009, η^2^ = 12.118, *r* = 0.8219). Statistical trends were revealed at region fronto-polar in theta-alpha (*Z* =  − 1.7838, *p* = 0.074) and alpha–beta-gamma (*Z* =  − 1.6818, *p* = 0.093), frontal-complete in alpha–beta-gamma (*Z* = , *p* = 0.059), and occipital in theta-alpha (*Z* =  − 1.8857, *p* = 0.059) (Fig. 2).

**Fig. 3:**
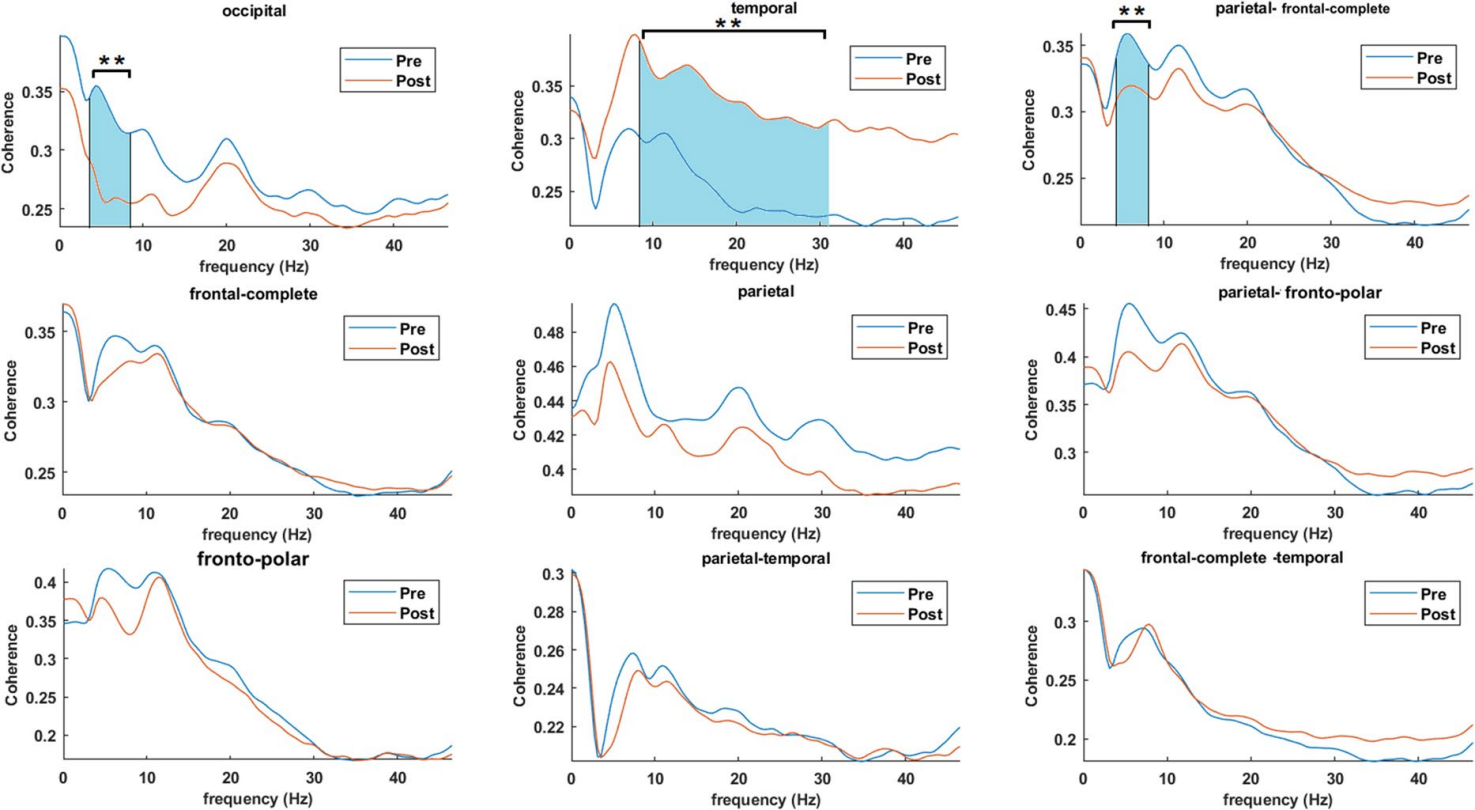
Effect of one TPS session on coherence. Grand average coherence (across patients and channels) corresponding to regions occipital, temporal, parietal-frontal-complete, frontal-complete, parietal, parietal-fronto-polar, fronto-polar, parietal-temporal, and frontal-complete-temporal. Significant differences between conditions are indicated by the shadowed regions. Figure marked with significant differences (**)

We found a significant difference in coherence between condition pre- and post- TPS at region occipital in theta (*Z* = 2.2934, *p* = 0.021, η^2^ = 3.74, *r* = 0.7252), and temporal in both alpha (*Z* =  − 2.2934, *p* = 0.021, η^2^ = 11.455, *r* = 0.7252) and beta (*Z* =  − 1.9876,*p* = 0.047, η^2^ = 10.809, *r* = 0.6285) as well as parietal-frontal-complete in theta (*Z* = 1.9876, *p* = 0.047, η^2^ = 4.124, *r* = 0.6285) (Fig. 3).

**Fig. 4:**
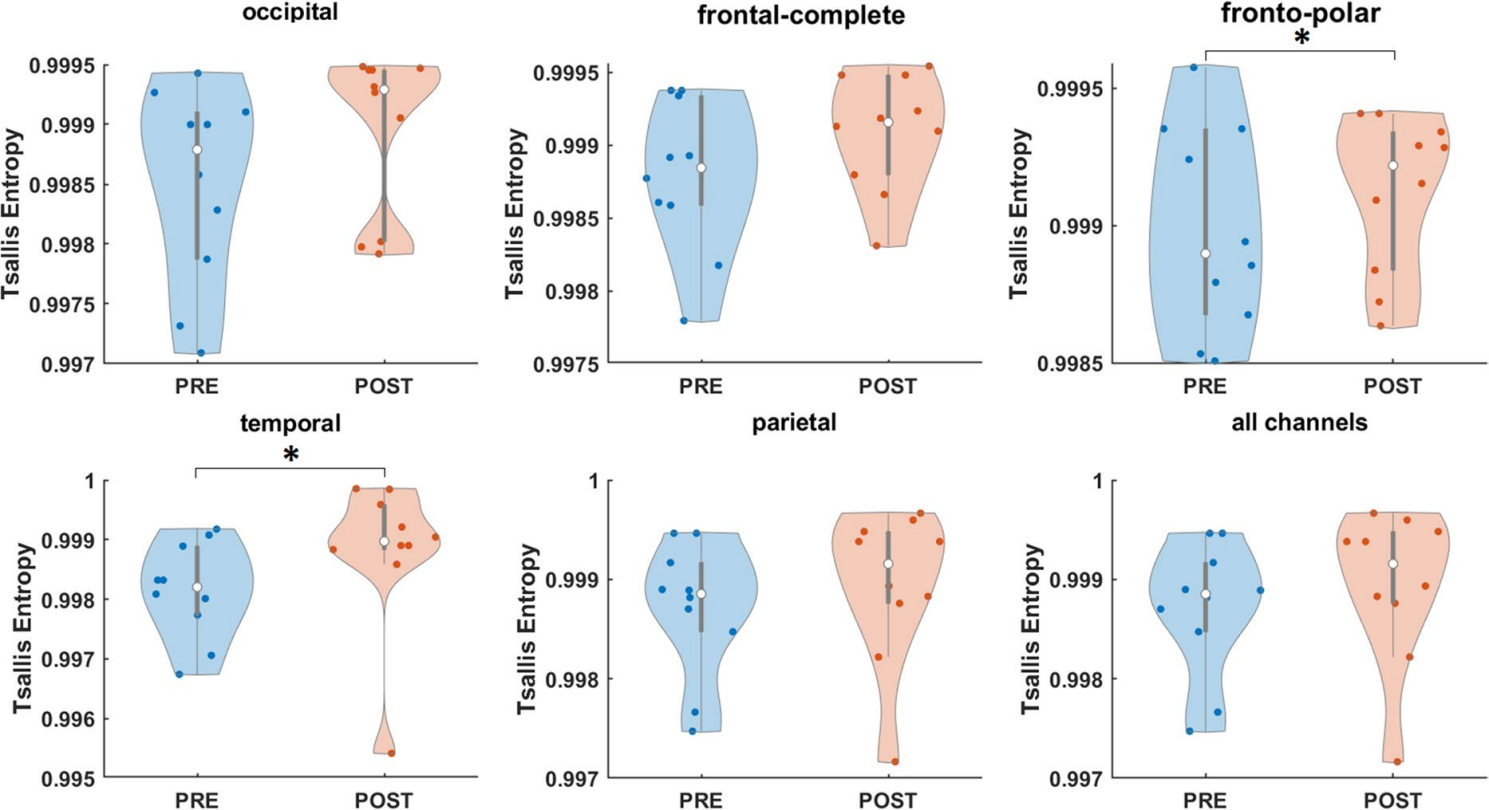
Effect of one TPS session on Tsallis entropy (TE). Individual effects (across channels) at occipital, frontal-complete, fronto-polar, temporal, parietal, and all channels. Figure marked with statistical trends (*)

Focusing on TE, Wilcoxon signed-rank test revealed a statistical trend in the difference between condition pre- and post-TPS at region temporal (*Z* =  − 1.7849, *p* = 0.074) and fronto-polar (*Z* =  − 1.8869, *p* = 0.059, η^2^ = 10.598, r = 0.5967) (Fig. 4).

**Fig. 5:**
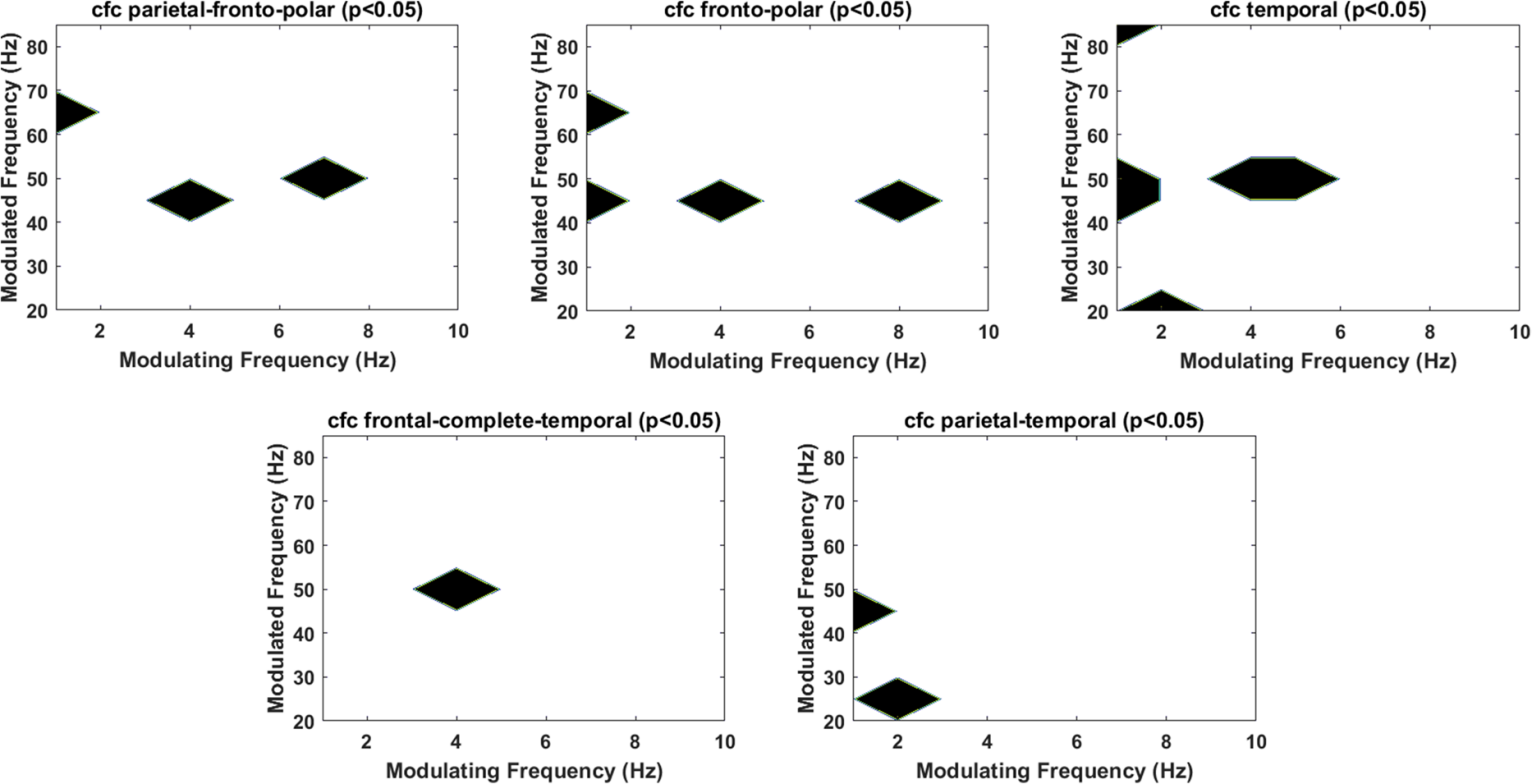
Effect of TPS on cross-frequency coupling (cfc) corresponding to regions parietal- fronto-polar, temporal, fronto-polar, temporal, frontal-complete-temporal, and parietal-temporal. Frequencies in which there is a significant cfc difference between pre and post TPS are highlighted in black for each considered region. Note that frequencies in the *x*-axis depict the modulating frequency within the range [1, 10 Hz] and those in the *y*-axis refer to the modulated frequency [20, 85 Hz]

Wilcoxon signed-rank test revealed significant differences between condition pre- and post-TPS in cfc at region fronto-polar (delta/theta-gamma (*Z* = 1.9876, 2.1915, 1.9876, *p* = 0.047, 0.029, 0.047, η^2^ = 4.124, 3.866, 4.124, *r* = 0.629, 0.693, 0.629), alpha-gamma (*Z* = 1.9876 *p* = 0.047, η^2^ = 4.124, *r* = 0.629)) and temporal (delta/theta-gamma (*Z* = 2.0896, 1.9876, 2.0896, 2.0896, *p* = 0.037, 0.047, 0.037, 0.037, η^2^ = 3.994, 11.674, 3.994, 3.994, *r* = 0.660, 0.629, 0.660, 0.660), delta-beta (*Z* =  − 1.9876, p = 0.047, η^2^ = 10.809, *r* = 0.629)). We found significant differences between condition pre- and post-TPS in cfc between parietal and fronto-polar (delta/theta-gamma (*Z* = 1.9876, 2.0896, 1.9876, *p* = 0.047, 0.037, 0.047, η^2^ = 4.124, 3.994, 4.124, r = 0.6285, 0.6608, 0.6285)), frontal-complete and temporal (theta-gamma (*Z* = 2.0896, p = 0.037, η^2^ = 3.994, *r* = 0.6608)), and parietal and temporal (delta-gamma\beta (*Z* = 2.0896, − 1,9876, *p* = 0.037, 0.047 η^2^ = 3.994, 10.809, *r* = 0.6608, 0.6285)) (Fig. 5).

## Discussion

Given the novelty of TPS, our study is exploratory in nature and thus it is primarily intended to generate data-driven hypotheses on the neurophysiological underpinnings of TPS in AD rather to confirm a particular hypothesis. As such, we emphasize the descriptive aspect of our results rather than the comparative aspect; consequently, multiple comparisons are not emphasized. Note that the importance of exploratory studies as a crucial step towards better hypothesis-driven confirmatory research has been stressed particularly in the field of neuromodulation [25]. As indicated in [26], neurodegeneration and functional changes in brain regions such as the dorsolateral prefrontal cortex may cause that AD patients are no longer able to retain the information needed to produce a placebo effect. As such, we expected that the neurophysiological effects reported here are less likely to be influenced by a placebo effect.

**Fig. 6:**
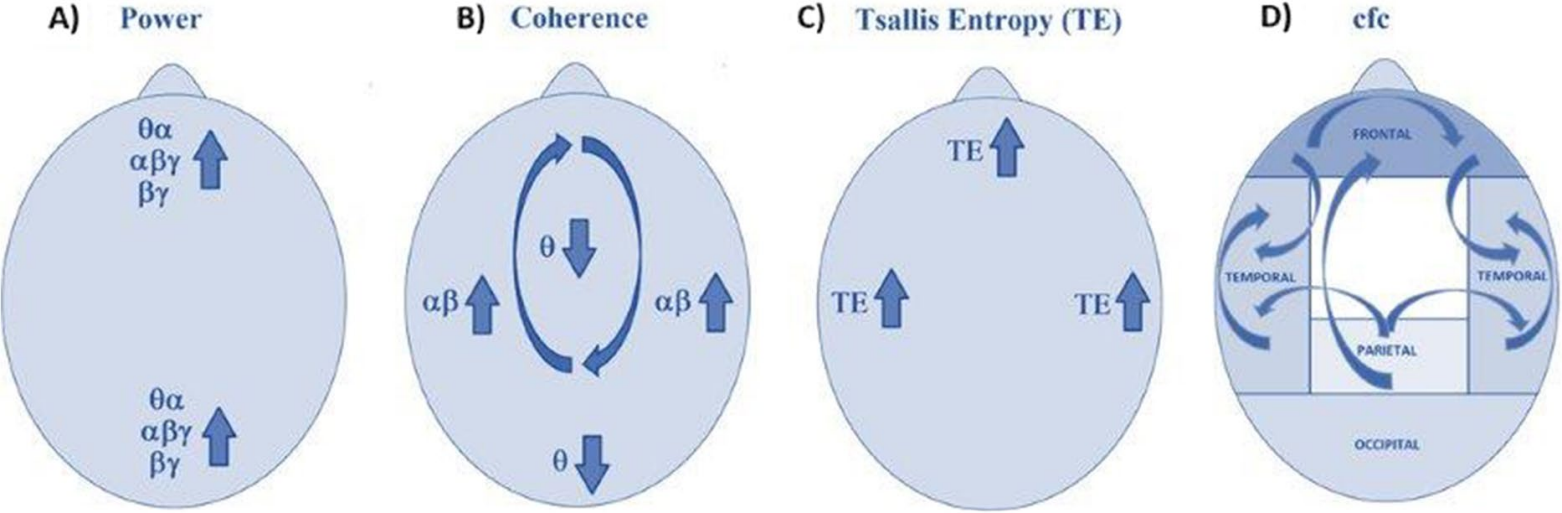
Summary of neurophysiological effect of one session of TPS. **A** Increase in power at occipital and frontal (theta-alpha, alpha–beta-gamma, and beta-gamma); **B** increase in coherence at temporal (alpha–beta) and decrease at occipital and parietal-frontal complete (theta); **C** increase in Tsallis entropy (TE) at fronto-polar and temporal regions;** D** changes in cross-frequency coupling (cfc) (parietal-fronto-polar, fronto-polar, temporal, frontal-complete-temporal, parietal-temporal)

Figure 6 summarizes our findings about the neurophysiological effect of one session of TPS in AD by focusing on (A) power, (B) Coherence, (C) TE, and (D) cfc. Note that the indicated effects emphasize the cortical areas of interest addressed.

The significant changes and statistical trends in spectral power, coherence, TE, and cfc after one TPS session reported here might indeed reflect a direct biological effect by the stimulation itself on the brain. However, as stimulation was conducted with 4 Hz, effects on alpha-to-gamma oscillations do not necessarily mean that neuronal clusters were stimulated directly. An indirect effect is plausible through stimulation of mechanosensitive ion channels, increased metabolism, and the release of nitric oxide in the treated areas [18, 27].

Previous studies addressing visuo-spatial processing impairment in early AD patients [28] reported reduction in beta band functional connectivity in the prefrontal cortex, which was associated to poor planning of navigation strategies. Interestingly, our results indicate that one session of TPS was able to increase beta power and modulate the cross-frequency coupling at frontal regions, which may be indicative of a positive effect provided by the stimulation.

The observed increase in coherence at temporal regions in alpha and beta together with decrease in parietal-frontal in theta seems to be positive when considering previous studies reporting a reduction of alpha coherence between central and temporal regions [29].

TE is a measure of uncertainty that has particularly been proposed for instance to quantify the presence and extent of development of burst suppression in EEG following brain injury [30] and as a biomarker of dementia in AD as it is able to detect EEG abnormalities reflected as a reduction in TE with respect to a healthy cohort [31]. In line with this, our preliminary results indicate that TE was sensitive to one application of TPS in AD patients particularly at temporal and occipital regions. Moreover, TPS generally favored an increase in TE across different regions, which contrasts with previous studies proposing that reduced TE may be indicative of reduced information processing capacity in patients with neurodegenerative disorders such as Parkinson’s disease [32]; in our case, an increase of TE may be indicative of a positive effect.

One of the most distinctive patterns of neural activity is the occurrence of brain oscillations, which may result from the synchronized activity of neurons, also implicated in brain communication and processing at different spatial and temporal scales. In line with this, cfc represents a measure of the interaction between brain oscillations at different frequency bands. Here, we considered cross-frequency phase amplitude coupling, namely the coupling of a signal’s phase at a modulating frequency range and the amplitude at a modulated frequency range. Previous studies addressing cfc as a marker of disease progression in AD and mild cognitive impairment (MCI) patients reported lower gamma (modulated)/theta (modulating) cfc compared to healthy controls, thus suggesting that gamma/theta cfc is important for proper cognitive functioning and that a decrease in gamma/theta cfc may be a sign of disease progression [33]. In the present study, one session of TPS triggered significant changes in gamma/theta cfc at parietal-fronto-polar, fronto-polar, temporal, and frontal-complete-temporal regions.

Concerning the reported effects on gamma oscillations, it should be noted that this might go beyond pure functional electrical networks. It is described that increase of gamma power can be found after gamma-tACS in conjunction with decreased hippocampal beta-amyloid levels in AD [34]. Thus, TPS changes on gamma-networks should be observed with special interest as shock waves might affect the brains glymphatic clearance system.

To gain experience concerning tolerability and safety, the number of impulses and the areas were stepwise increased after the first session in the pilot patients. Thus, it should be considered a limitation of the study that stimulated areas differed in some patients from the complete protocol. However, a common ground in all patients was that parts of the default mode-network, especially the precuneus, were stimulated. That area is crucial for information processing in Alzheimer’s and has already been targeted in neuromodulation studies [8].

Limitations of the present study include also a small sample size, which affects the statistical power of our analysis. Our study is cross-sectional and provides neurophysiological effects of one session of TPS at one point in time. Future studies should consider the effects of TPS at different time points and under several sessions of TPS in relation to cognitive scores to clarify the prospect of EEG measures as neurophysiological biomarkers of TPS in relation to cognitive scores.

## Conclusion

Our results support the role of one session of TPS in modulating oscillatory activity and connectivity of electrical brain networks with potential implications in cognitive functioning and modulation of plasticity.

## Supplementary information

Below is the link to the electronic supplementary material.

Supplementary file1 (M 1 KB)

Supplementary file2 (M 1 KB)

## Data Availability

Data of this study are available from the corresponding author upon reasonable request.

## Abbreviations

AD: Alzheimer’s disease
TPS: Trancranial pulse stimulation
EEG: Electroencephalogram
TE: Tsallis entropy
cfc: Cross-frequency coupling
TMS: Trancranial magnetic stimulation
tDCS: Trancranial direct current stimulation
DBS: Deep brain stimulation
FUS: Focal ultrasound
